# Combined inhibition of Aurora A and p21-activated kinase 1 as a new treatment strategy in breast cancer

**DOI:** 10.1007/s10549-019-05329-2

**Published:** 2019-06-28

**Authors:** Vlad A. Korben, Michelle Borakove, Yayi Feng, William M. Wuest, Alex B. Koval, Anna S. Nikonova, Ilya Serebriiskii, Jonathan Chernoff, Virginia F. Borges, Erica A. Golemis, Elena Shagisultanova

**Affiliations:** 1 Department of Pathology and Cell Biology, Columbia University College of Physicians and Surgeons, New York, NY 10032 USA; 2 Molecular Therapeutics Program, Fox Chase Cancer Center, Philadelphia, PA 19111 USA; 3 University of Colorado Denver, Young Women’s Breast Cancer Translational Program, Aurora, CO 80045 USA; 4 Department of Chemistry, Emory University, Atlanta, GA 30322 USA; 5 Department of Chemistry, Temple University, Philadelphia, PA 19122 USA; 6 Kazan Federal University, Kazan, 420000 Russian Federation; 7 Cancer Biology Program, Fox Chase Cancer Center, Philadelphia, PA 19111 USA; 8 University of Colorado Denver, Department of Medicine, Division of Medical Oncology, 12801 East 17th Ave, Room 8118, MS 8117, Aurora, CO 80045 USA

**Keywords:** Breast cancer, Targeted therapy, Aurora A, AURKA, p21-Activated kinase 1, PAK1

## Abstract

**Purpose:**

The serine-threonine kinases Aurora A (AURKA) and p21-activated kinase 1 (PAK1) are frequently overexpressed in breast tumors, with overexpression promoting aggressive breast cancer phenotypes and poor clinical outcomes. Besides the well-defined roles of these proteins in control of cell division, proliferation, and invasion, both kinases support MAPK kinase pathway activation and can contribute to endocrine resistance by phosphorylating estrogen receptor alpha (ERα). PAK1 directly phosphorylates AURKA and its functional partners, suggesting potential value of inhibiting both kinases activity in tumors overexpressing PAK1 and/or AURKA. Here, for the first time, we evaluated the effect of combining the AURKA inhibitor alisertib and the PAK inhibitor FRAX1036 in preclinical models of breast cancer.

**Methods:**

Combination of alisertib and FRAX1036 was evaluated in a panel of 13 human breast tumor cell lines and BT474 xenograft model, with assessment of the cell cycle by FACS, and signaling changes by immunohistochemistry and Western blot. Additionally, we performed in silico analysis to identify markers of response to alisertib and FRAX1036.

**Results:**

Pharmacological inhibition of AURKA and PAK1 synergistically decreased survival of multiple tumor cell lines, showing particular effectiveness in luminal and HER2-enriched models, and inhibited growth and ERα-driven signaling in a BT474 xenograft model. In silico analysis suggested cell lines with dependence on AURKA are most likely to be sensitive to PAK1 inhibition.

**Conclusion:**

Dual targeting of AURKA and PAK1 may be a promising therapeutic strategy for treatment of breast cancer, with a particular effectiveness in luminal and HER2-enriched tumor subtypes.

**Electronic supplementary material:**

The online version of this article (10.1007/s10549-019-05329-2) contains supplementary material, which is available to authorized users.

## Introduction

The serine-threonine kinases Aurora A (AURKA) and p21-activated kinase 1 (PAK1) are frequently overexpressed in breast tumors and associated with aggressive tumor phenotypes and poor clinical outcomes [1–4]. AURKA controls centrosome maturation, timing of mitotic entry, assembly of the bipolar spindle, and chromosome alignment in metaphase [5]. AURKA overexpression occurs in over 90% of breast carcinomas [3, 5]. Increased AURKA activity overrides the mitotic spindle assembly checkpoint, inducing resistance to anti-mitotic agents [6], while inhibition of AURKA increases the activity of microtubule inhibitors [7, 8]. In interphase, overexpressed AURKA stabilizes C-MYC [9] and stimulates the PI3K/AKT/mTOR pathway, promoting chemotherapeutic resistance [10].

Increased PAK1 activity is also common in breast cancer, typically due to amplification of the *PAK1* gene (30% of breast carcinomas) [11]. Like AURKA, PAK1 stimulates multiple pro-oncogenic pathways, including AKT, C-MYC, and β-catenin [11, 12], promoting proliferation, motility, and invasion [11, 13]. PAK1-dependent upregulation of cyclin D1 is important for G1/S transition [14]. Although AURKA and PAK1 function within overlapping but distinct signaling pathways, PAK1 is capable of AURKA activation: both directly, by phosphorylating serine S342 and threonine T288 in the activation loop [15], and indirectly, by phosphorylation of the AURKA-activating protein partners LIMK1 and ARPC1b [15–17].

Of relevance to breast cancer, both AURKA and PAK1 phosphorylate estrogen receptor alpha (ERα) (on serines S118 (PAK1) and S305 (PAK1 and AURKA)) supporting ligand-independent transcription of ERα-dependent genes promoting proliferation, invasion, and endocrine resistance [4, 18]. The AURKA inhibitor alisertib synergized with tamoxifen in preclinical studies [4] and showed activity in patients with hormone receptor-positive (HR +) breast cancer [19]. PAK1 inhibition has been reported to abrogate tamoxifen resistance [20].

Based on these activities of AURKA and PAK1, we hypothesized that combined inhibition of both could have synergistic anti-tumor effects in breast cancer [13, 21]. In this study, we explored the consequences of combination treatment with the alisertib and FRAX1036, a highly selective inhibitor of PAK1 and two paralogous group 1 PAK kinases, PAK2 and PAK3 [11].

## Materials and methods

See “Supplementary Materials” for additional details on cell lines, cell culture, antibodies for Western blot and IHC, drug formulations for xenograft experiments, and statistical analysis.

### Tumor cell lines, media, and reagents

Human breast cancer cell lines from the American Type Culture Collection were cultured in standard conditions. We confirmed negative mycoplasma testing and STR profile for each cell line. Alisertib was purchased from MedChem Express (Monmouth Junction, NJ). FRAX1036 was synthesized by AK and WW [22].

### Cell viability assay

Cells were grown on 96-well plates for 24 h before treatment with drug(s) or vehicle. Cell viability was measured by CellTiterGlo assay (Promega, Madison, WI) after 72 h of treatment. Each drug concentration was evaluated in triplicate, with ≥ 3 biological repetitions. We determined synergy by Chou-Talalay method [23].

### Western blotting

Protein lysates were prepared with RIPA lysis buffer (Thermo Fisher, Waltham, MA) containing protease/phosphatase inhibitor (Roche Diagnostic, Indianapolis, IN). Each blot was repeated with ≥ 3 preparations of lysates. Signal intensity was quantified by NIH ImageJ Software, or Odyssey imager software (Li-Cor Bioscience, Lincoln, NE), normalized to vinculin or GAPDH, and compared by two-tailed *t* test and one-way ANOVA.

### Xenograft studies

All animal experiments were approved by the FCCC Institutional Animal Care and Use Committee. NOD.Cg-Prkdc^scid^ Il2rg^tm1Wjl^/SzJ (NSG) mice from the FCCC breeding colony were maintained under pathogen-free conditions. Estrogen pellets were implanted subcutaneously into 6- to 8-week-old mice as described [24]; simultaneously, mice were injected in mammary fat pads with 10^7^ BT474 cells (*N* = 45 mice). Treatment consisted of alisertib (15 mg/kg twice a day), FRAX1036 (20 mg/kg daily) or combination of drugs; control group received vehicle solution twice a day; all agents were administered by oral gavage.

To assess short-term signaling, after tumor volume reached 600 mm^3^, mice were treated for 3 days with vehicle, alisertib, FRAX1036, or combination of drugs, then euthanized and tumors were frozen for Western blots. To assess long-term responses, once tumors reached 150 mm^3^, mice were treated for 21 days, then euthanized, and tumors collected for analysis.

### Immunohistochemistry (IHC)

IHC was performed according to standard protocols. Results were quantitated with Aperio ePathology (Leica Biosystems, Buffalo Grove, IL) and analyzed by Mann–Whitney and Kruskal–Wallis tests.

### Cell cycle analysis by fluorescence-activated cell sorting (FACS)

Non-synchronized growing cells were fixed with ethanol at 24 and 72 h after treatment with drug(s) or vehicle, then mixed with propidium iodide solution (BD Pharmingen, San Diego, CA) before FACS (BD Biosciences, San Diego, CA); data were analyzed by one-way ANOVA.

### In silico analysis of expression and zGARP scores for the genes of interest and correlation with FRAX1036 and alisertib activity in vitro

Methods for deriving z-score normalized Gene Activity Ranking Profile (zGARP) score have been described in detail [25, 26]. zGARP scores for *AURKA, CCND1, MYC, PAK1*-*3*, and *TFF1* were extracted from [25]. For *PAK1*-*3*, we selected the smallest of the zGARP scores for each cell line. RNAseq fragments per kilobase million (FPKM) values were extracted from [25, 27–29]. For each gene, ranks were calculated across cell lines indicated in Results in each dataset. Ranks for gene/cell line pairs were averaged across the sets of RNAseq data. Pearson correlation coefficients and *p* values were calculated using GraphPad Prism for the drug IC50 versus zGARP score.

## Results

### Alisertib and FRAX1036 synergize predominantly in luminal and HER2-enriched breast cancer cell lines

**Fig. 1:**
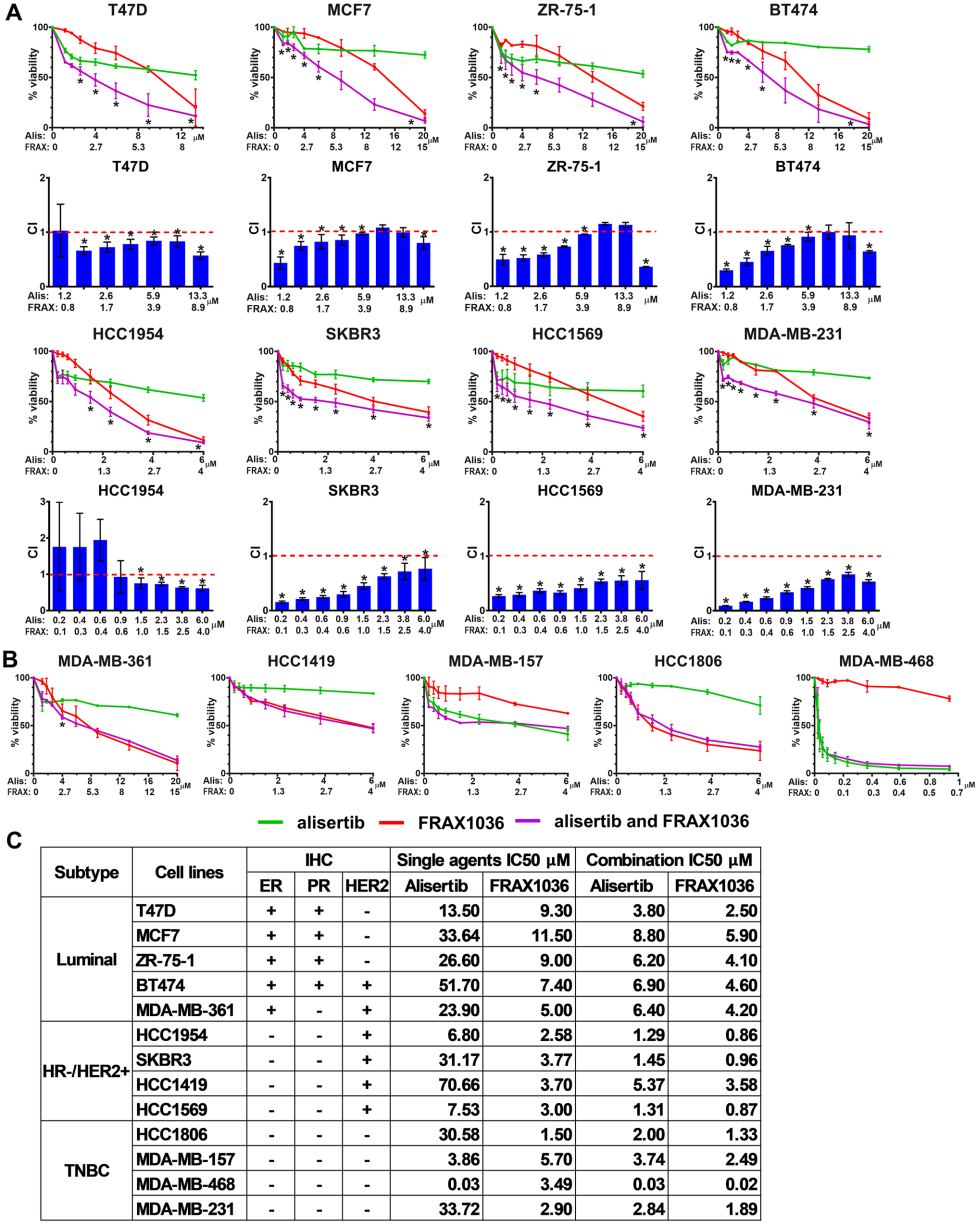
Cell viability in breast cancer cell lines treated with FRAX1036 and alisertib. **a**, **b** X-axis, concentration of alisertib (Alis) or FRAX1036 (FRAX) in µM, with all experiments conducted at a constant molar ratio of alisertib:FRAX1036 at 1.5:1. **a** Cell lines with demonstrated synergy of alisertib/FRAX1036 combination; drug concentrations that showed synergy are marked with asterisks; Chow-Talalay analysis of synergy is presented below each cell viability graph (CI—combination index; CI < 1 indicate synergy, CI = 1 additive effect; CI > 1 antagonistic effect). **b** Cell lines without demonstrated synergy. **c** Expression profile for estrogen receptor (ER), progesterone receptor (PR), and HER2 in the cell lines assessed (as published in Marcotte et al. [25], and Gazdar et al. [51]) as well as IC50 (in µM) for alisertib and FRAX1036 used as single agents and in 1.5:1 combination ratio

We evaluated the effect of dual inhibition of AURKA and PAK1 on the proliferation of 5 luminal (MCF7, ZR75, T47D, BT474, MDA-MB-361), 4 hormone receptor negative (HR-) human epidermal growth factor receptor 2 positive (HER2 +) (HCC1954, HCC1419, HCC1569, SKBR3), and 4 triple negative (TNBC) (MDA-MB-157, MDA-MB-468, MDA-MB-231, HCC1806) breast cancer cell lines (Fig. 1, S1). Single agent alisertib had low (0.03 and 3.86 μM) IC50 values in 2 of 4 TNBC cell lines (MDA-MB-468 and MDA-MB-157), but higher values in 2 other TNBC lines, and all luminal and HR-/HER2 + cell lines. Single agent FRAX1036 was active in HR-/HER2 + cell lines (IC50 2.6–3.8 µM) and TNBC cell lines (IC50 1.5–5.7 µM), but less so in luminal cell lines (IC50 5.0–11.5 µM).

Considering the maximum tolerated doses of alisertib and FRAX1036 in vivo [30, 31] and clinically relevant doses of alisertib in humans [32, 33], we selected a fixed molar ratio of FRAX1036 to alisertib of 1:1.5 for assessment in cell lines (Fig. 1, S1). Synergy between alisertib and FRAX1036 was detected in four of five luminal cell lines, particularly at lower drug concentrations (Fig. 1, S2); activity of alisertib and FRAX1036 combination exceeded efficacy of fulvestrant in these cell lines (Fig. S3). Alisertib and FRAX1036 also synergized in 3 of 4 HR-/HER2 + tumor cell lines, but only in 1 of 4 TNBC cell lines (Fig. 1, S2).

### Alisertib and FRAX1036 change cell cycle compartmentalization and decrease activity of ERα and MYC in tumor cell lines

**Fig. 2:**
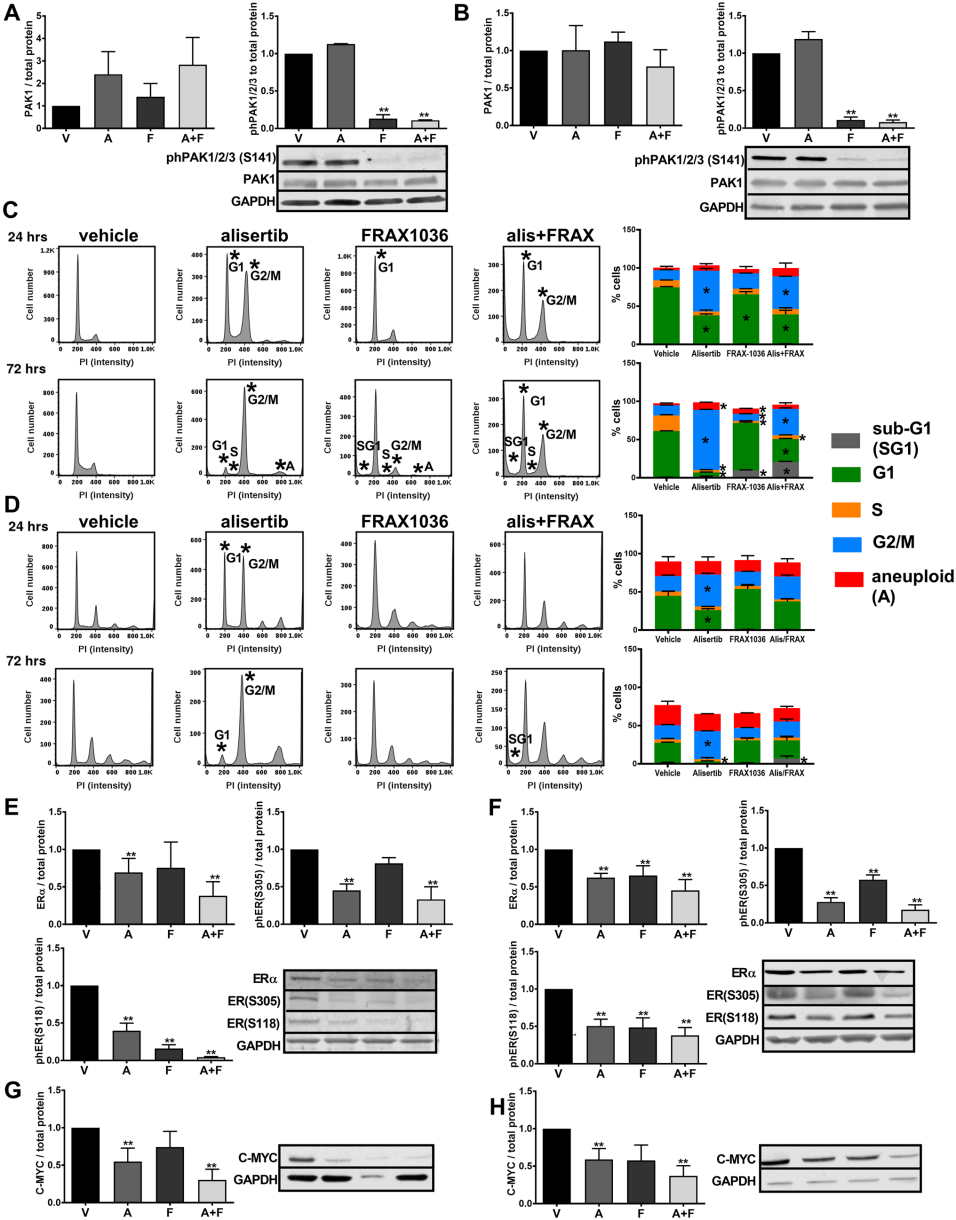
Alisertib and FRAX1036 are active in T47D and BT474 cells in vitro. Data shown indicate analysis of T47D or BT474 cell lines treated with alisertib and FRAX1036 or combination at IC30 for 72 h prior to collection of protein lysates for Western blotting and for 24 and 72 h prior to FACS analysis. **a, b** Western blot visualization of phosphorylated PAK1/2/3 and total PAK1 kinase in BT474 (**a**) and T47D (**b**) cell lines. **c, d** Cell cycle compartmentalization, quantification, and representative data for BT474 (**c**) and T47D (**d**) cell lines; asterisks mark significant differences (*p* ≤ 0.05) in the proportion of cells between treatment groups versus vehicle by one-way ANOVA. **e, f** Combination of FRAX1036 and alisertib suppressed phosphorylation of ERα(S305) and ERα(S118) in BT474 (**e**) and T47D (**f**) tumor cell lines. **g, h** Combination of alisertib and FRAX1036 suppressed expression of C-MYC in BT474 (**g**) and T47D tumor cell lines (**h**); V—vehicle; F—FRAX1036; A—alisertib; A + F—alisertib and FRAX1036 combination; double asterisks mark *p* ≤ 0.05 relative to vehicle by two-tailed *t* test

Because FRAX1036 and alisertib were most active in luminal and HER2 + cell lines, we selected the T47D (HR +/HER2-) and BT474 (HR +/HER2 +) cell lines for evaluation of cell cycle and signaling changes upon co-inhibition (Fig. 2). Both FRAX1036 and the combination treatment effectively and significantly reduced phospho-PAK1/2/3 in BT474 and T47D tumor cell lines (Fig. 2a, b). No antibody effectively detected endogenous phospho-AURKA(T288) by Western blot [5], prohibiting parallel analysis. However, alisertib caused characteristic G2/M arrest in both cell lines, providing an independent measure of substantial AURKA inhibition after 24 or 72 h of treatment (Fig. 2c, S4, S5). The degree of G2/M arrest exceeded inhibition of cell viability induced by alisertib in these cell lines (Fig. 1), likely because the arrest did not lead to cell death immediately, but was predominantly cytostatic over a short treatment time in vitro. In BT474, we observed an alisertib-induced increase in aneuploid (> 4 N) cells, reflecting the inability of cells to progress effectively through cytokinesis. In BT474, FRAX1036 induced G1 arrest, with subsequent increase in sub-G1 and > 4 N cells, and decrease in S-phase and G2 M cells at 72 h. Treatment of BT474 cells with alisertib/FRAX1036 combination resulted in accumulation in sub-G1, G1 and G2/M populations, with a decreased proportion of cells in S-phase (Fig. 2c, S4). Combination treatment also caused accumulation of > 4 N and sub-G1 populations in the T47D cell line, particularly by 72 h of treatment (Fig. 2d, S5). Additionally, alisertib or combination treatment led to significant inhibition of phosphorylation of the pathognomonic AURKA substrate Polo-like kinase 1 (PLK1), confirming specificity of targeted inhibition (Fig. S6).

Notably, the drug combination significantly inhibited phosphorylation of ERα(S118) and ERα(S305) in both cell lines (Fig. 2e, f). Alisertib and FRAX1036 also inhibited phosphorylation of ERα(S118) in both cell lines, although to a lesser degree than the combination. Expression of C-MYC was reduced more by the alisertib/FRAX1036 combination than by single agents in both lines (Fig. 2g, h).

### Activity of combined versus monoagent alisertib and FRAX1036 in BT474 tumor xenografts

**Fig. 3:**
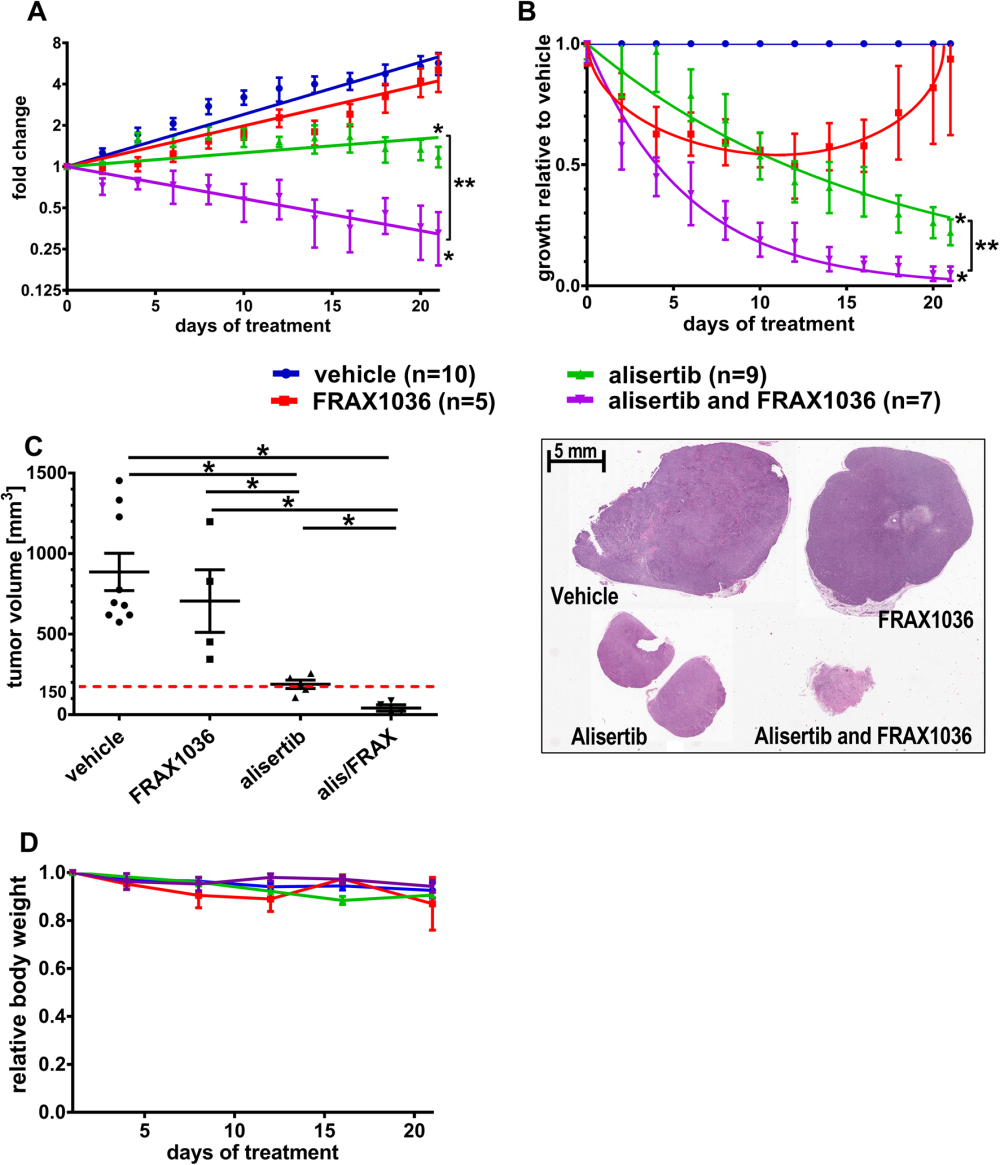
Inhibition of AURKA and PAK1 limits growth of BT474 mammary xenograft tumors. Tumor volumes were approximated as length × width^2^ × 0.52. Data presented as tumor volume at the point of time relative to the tumor volume at the initiation of treatment (**a**) or tumor volume at the point of time relative to vehicle-treated group, mean ± SEM with non-linear regression curve fit (**b**). Regression analysis for alisertib versus vehicle, alisertib versus FRAX1036, combination versus vehicle, and combination versus FRAX1036 was significant with *p* < 0.001 (marked with asterisks); combination versus alisertib—*p* = 0.003 (marked with double asterisk) and *p* value for synergy of the combination—*p* = 0.014. **c** Absolute tumor volumes at the end of treatment; dotted red line represents tumor volume at the start of treatment; asterisks mark *p* ≤ 0.05 by two-tailed *t* test; representative tumors from mice treated with vehicle, alisertib, FRAX1036, and the alisertib/FRAX1036 combination groups are shown. **d**. Changes in the weight of the mice on treatment relative to the initial weight, data presented as mean ± SEM

We evaluated the drug combination in vivo using BT474 (HR +/HER2 +) orthotopic xenografts. Tumors were established in NOD/SCID mice and treated for 21 days with vehicle, FRAX1036 20 mg/kg, alisertib 15 mg/kg, or combined FRAX1036/alisertib (Fig. 3).

By regression analysis, reduction in the BT474 tumor growth rate compared to vehicle was significant in mice treated with alisertib or alisertib/FRAX1036 (*p* < 0.001), but not in FRAX1036-treated mice (Fig. 3a, b). Tumor control with combination therapy was better than with monotherapy (*p* < 0.001 combination versus FRAX1036, *p* = 0.003 combination versus alisertib, *p* value for synergy *p* = 0.014). Although FRAX1036 produced initial responses, they were lost after 10 days (Fig. 3b). Considering the difference of FRAX1036 activity in vivo and in vitro, tumor microenvironment likely plays a strong role in resistance mechanisms, based on emerging understanding of PAK function [34]. After 21 days, tumor volume averaged 930 mm^3^ in vehicle-treated mice, 826 mm^3^ in FRAX1036-treated mice, 188 mm^3^ in alisertib-treated mice, and 55 mm^3^ in mice treated with the combination (Fig. 3c). Final tumor volumes differed significantly between the alisertib or the combination versus vehicle (*p* < 0.05); further, tumor volume with the combination treatment was smaller comparing to monagent alisertib (*p* = 0.004). Importantly, only the alisertib/FRAX1036 combination reduced tumor volume compared to the initial volume (~ 150 mm^3^) (Fig. 3c), with histopathological analysis indicating one case of near complete response (residual tumor volume of 16 mm^3^) and one case of complete response in treated mice. All therapies were well tolerated, with weight of drug- and vehicle-treated mice not significantly differing (Fig. 3d).

### Immunohistopathological (IHC) assessment of xenografts

**Fig. 4:**
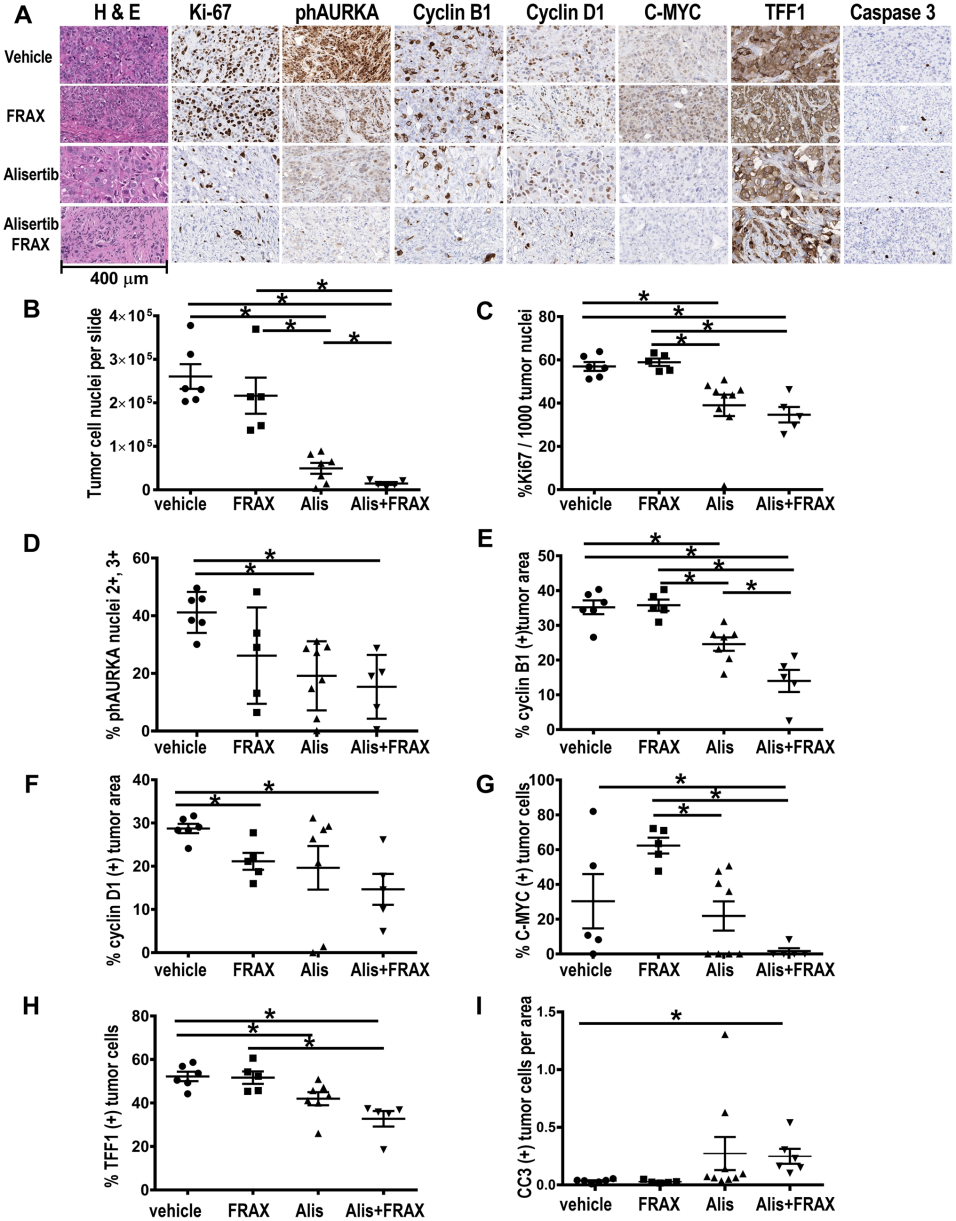
Immunohistochemistry of BT474 xenografts. **a** Representative tumor sections for quantified data. **b** Tumor nuclei count per slide. **c** Percentage of tumor cell nuclei positive for Ki-67. **d** Percentage of tumor cell nuclei strongly positive for phospho-AURKA. **e** Percentage of tumor area positive for cyclin B1. **f**–**h** Percentage of tumor cell nuclei positive for cyclin D1 (**f**), C-MYC (**g**), or TFF1 (**h**). **i** Percentage of cleaved caspase-3 (CC3) positive tumor cells per tumor area. Mice that completed ≥ 14 days of treatment were included in analysis; asterisks indicate *p* ≤ 0.05 by Mann–Whitney test

Xenograft tumors were analyzed by IHC (Fig. 4). The fewest cancer cells and the largest areas of fibrosis and necrosis were found in tumors treated with the combination (Fig. 4a). Monoagent alisertib or FRAX1036 also increased fibrotic areas in tumors, albeit to a lesser degree than the combination. The significantly reduced tumor cellularity found with the combination therapy (Fig. 4b) suggested a greater treatment effect than that indicated by solely considering average residual tumor volume. In residual tumor cells, treatment with alisertib or alisertib/FRAX1036 significantly decreased expression of the Ki67 proliferation marker (Fig. 4a, c). Phosphorylation of AURKA was significantly decreased by alisertib, and to a greater extent by combination treatment (Fig. 4a, d).

To better characterize treatment-induced cell cycle arrest, we evaluated cyclin D1, and the mitotic cyclin B1. Alisertib significantly reduced cyclin B1 expression (Fig. 4a, e), consistent with the requirement of AURKA for G2/M transition [35]. FRAX1036 significantly decreased cyclin D1 expression (Fig. 4a, f), reflecting the essential role of PAK1 in induction of this gene [14]. Combination therapy reduced expression of both cyclins to a much greater extent than with either single agent, suggesting quiescent or moribund cells (Fig. 4a, e, f).

C-MYC [36] and trefoil factor 1 (TFF1) [37] are canonical downstream effectors of ERα. After 3 weeks of treatment, all tumors treated with combination therapy had very low to undetectable expression of C-MYC, which was significantly different from the control or single agents (Fig. 4a, g). In contrast, FRAX1036 numerically increased C-MYC levels versus all other treatment groups, suggesting a rebound effect and potential escape mechanism. Combination therapy significantly decreased TFF1 expression, with a more modest reduction seen in single agent alisertib-treated tumors (Fig. 4a, h). Expression of the apoptotic marker cleaved caspase-3 (CC3) was increased in tumors treated with the combination of alisertib and FRAX1036 compared to control vehicle-treated cells (Fig. 4a, i). However, the number of CC3 positive cells was small, potentially indicating alternative mechanisms of cell death are also involved, such as necrosis, mitotic catastrophe, or senescence. In sum, these results indicated functional activity of combined alisertib/FRAX1036 in xenografts, reflected in decreased tumor volume, reduced cellularity, suppressed Ki67, altered cell cycle checkpoints, and depressed ERα signaling.

### Alisertib and FRAX1036 inhibit PAK1 and ERα signaling following transient treatment of BT474 tumors in vivo

**Fig. 5:**
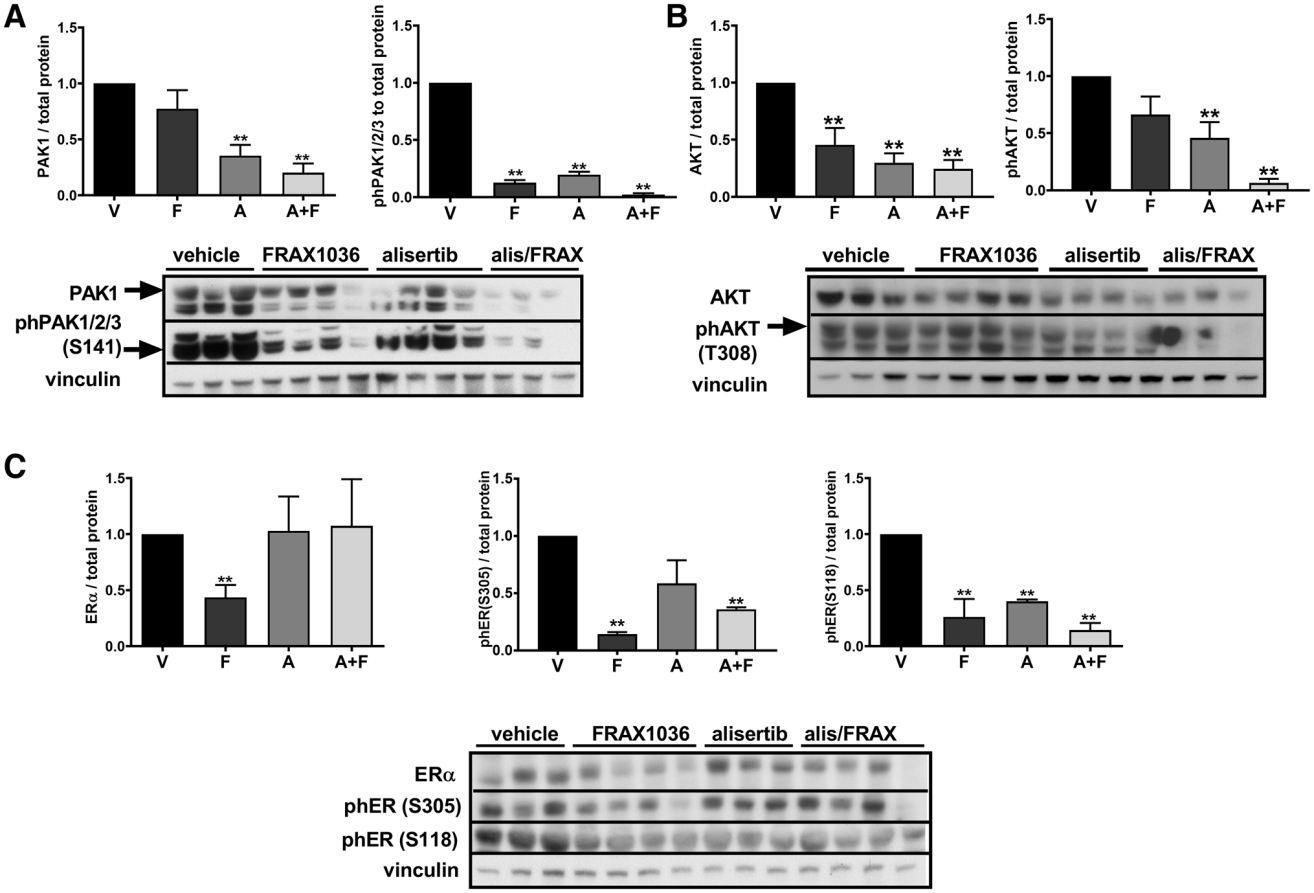
Consequences of alisertib and FRAX1036 treatment for PAK and ERα phosphorylation in BT474 xenograft tumors. Western blot of BT474 xenografts dosed for 3 days with indicated drugs. Changes in the total and phospho-PAK kinase (**a**), total and phospho-AKT (**b**), total ERα and phosphorylated ERα(S305) and ERα(S118) (**c**). V—vehicle; F—FRAX1036; A—alisertib; A + F—alisertib and FRAX1036 combination; double asterisks mark *p* ≤ 0.05 relative to vehicle by two-tailed *t* test

To explore the short-term effect of our drugs, we established BT474 xenografts (*n* = 3–4 per treatment group) and treated mice with vehicle, alisertib, FRAX1036, or the combination for 3 days, then analyzed tumor lysates. FRAX1036 effectively reduced levels of phospho-PAK1/2/3 (Fig. 5a). Alisertib also resulted in decreased phospho-PAK1/2/3, likely via inhibition of phospho-AKT (Fig. 5b) [10, 38]. The drug combination nearly completely eliminated PAK and AKT phosphorylation (Fig. 5a, b). Treatment with FRAX1036 reduced total ERα, while FRAX1036 and the combination reduced phosphorylation of ERα(S305), and treatment with monoagents or drug combination suppressed phosphorylation of ERα(S118) (Fig. 5d).

### Differential response to alisertib and FRAX1036 correlates with AURKA and MYC zGARP scores

**Fig. 6:**
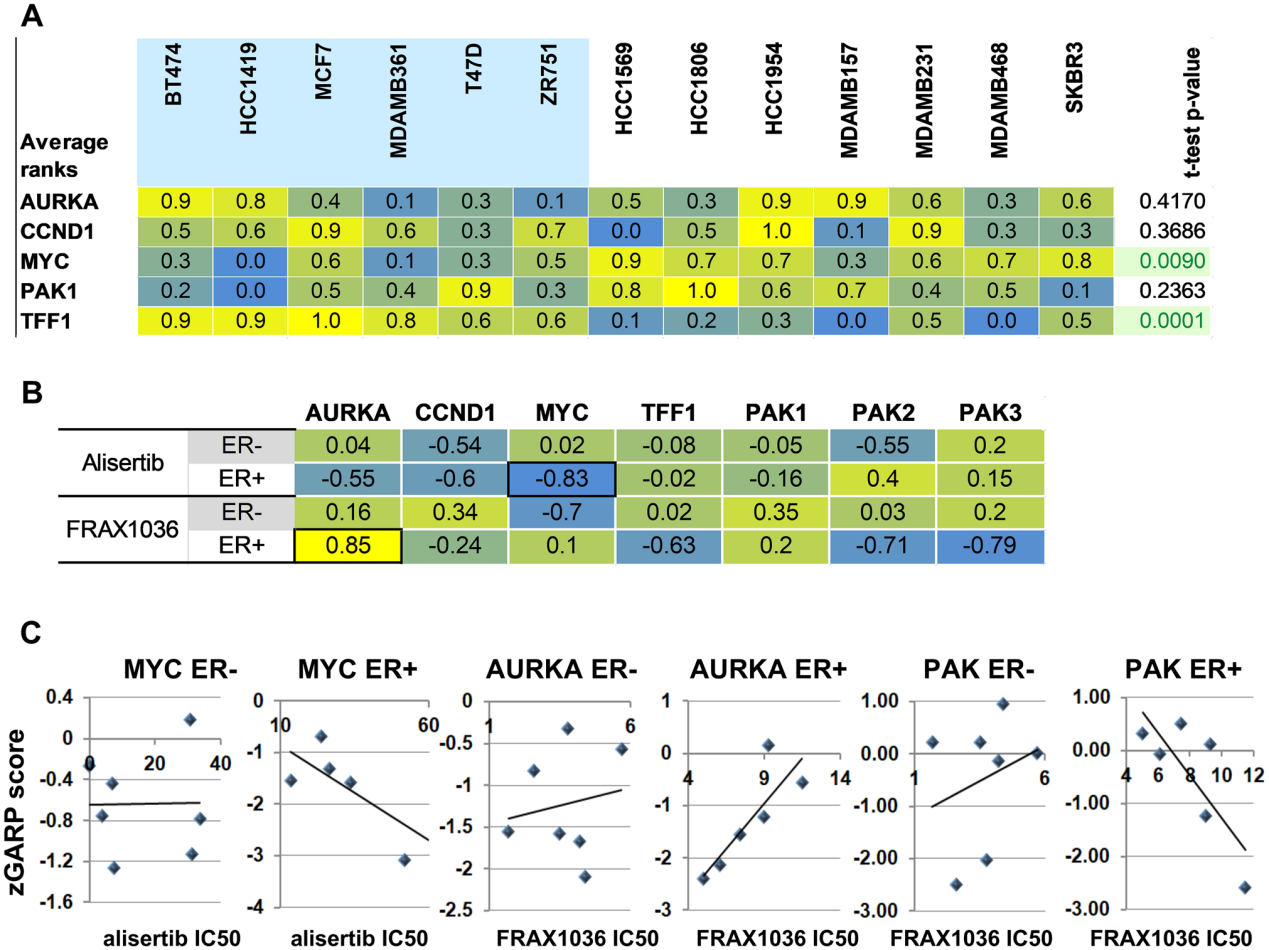
mRNA expression levels, sensitivity to shRNA-mediated knockdown and to the alisertib/FRAX1036 treatment in the tested cell lines. **a** Relative expression of indicated genes in the set of cell lines used in this study. RNAseq fragments per kilobase million (FKMP) values were extracted from studies [25, 27–29]. For each gene, the rank level of expression was calculated across the set of cell lines, with 1.0 indicating the highest expression level in the set and 0.0 indicating the lowest. Average ranks are shown. ERα positive cell lines are shaded in blue; two-tailed *t* test was used to assess the significance in expression differences between of ERα + and ERα- cell lines. **b** zGARP scores for *AURKA*, *CCND1*, *MYC*, *PAK1*-*3* and *TFF1* versus cell line sensitivity to alisertib (top) and FRAX1036 (bottom). The lower the zGARP score, the more essential the gene is for tumor survival. Pearson correlation coefficients were calculated for ER + and ER− cell lines; significant (*p* ≤ 0.05) correlations are indicated with black borders. **c** Differential correlation of *MYC*, *AURKA*, and *PAK* zGARP scores with drug sensitivity indicates dependence on ER status. For *PAK1*-*3*, the lowest of the zGARP scores for each cell line was used. See text for details

To gain further insight into parameters associated with response to drug treatment in vitro, we explored several comprehensive datasets reporting gene and protein expression in breast cancer cell lines [25, 27–29]. We analyzed *AURKA*, *PAK1,* and a group of functionally related genes with expression known to be regulated by ERα, including cyclin D1 (*CCND1*), *C*-*MYC*, and *TFF1*. Integration of four RNAseq datasets confirmed the expected higher expression of *C*-*MYC* and *TFF1* in ERα + versus ERα- subsets (Fig. 6a). No significant differences were found in the expression of *AURKA, PAK1,* and *CCND1* based on ERα status. There was no correlation between the drug response to alisertib or FRAX1036 and the pretreatment expression levels of these genes, either at mRNA or protein levels, for members of a broad panel of ERα + or ERα- cell lines.

A database of gene essentiality in tumor cell lines has been determined by shRNA knockdown and characterized by z-score normalized Gene Activity Ranking Profile (zGARP) score [25, 26]. The zGARP score reflects changes in gene expression and cellular proliferation after treatment of tumor cells with shRNAs [26]. Response to a targeted agent may correlate with gene essentiality even if it does not correlate with gene expression [25]. We correlated zGARP scores for *AURKA*, *PAK1*-*3*, *CCND1*, *C*-*MYC*, and *TFF1* with response to alisertib and FRAX1036 in our cell line experiments (Fig. 6b, c). In ERα + lines, the strongest predictor of response to alisertib was the strength of dependence on *C*-*MYC*, a relationship not observed in ERα- lines (Fig. 6b, c). ERα + cell lines highly sensitive to shRNA *C*-*MYC* knock down required higher concentrations of alisertib for growth inhibition, compared to less dependent cell lines. Weaker, but suggestive relationships with alisertib response in ERα + lines were found for dependence on *CCND1* and the alisertib target, *AURKA* (Fig. 6b). Similar analysis performed for FRAX1036 (Fig. 6b, c) revealed correlation with dependence on *PAK2* and *PAK3*, both of which are FRAX1036 targets along with *PAK1*, as well as weaker correlation with dependence on *TFF1* (Fig. 6b, c). Intriguingly, the strongest interrelationship found was positive correlation of sensitivity to FRAX1036 with dependence on *AURKA* in ERα + cell models, suggesting that cells with strong requirements of *AURKA* might be more sensitive to PAK inhibition (Fig. 6b, c). Because zGARP scores were developed to predict individual drug sensitivity [28], we did not analyze correlation of zGARP scores with the efficacy of two drugs in combination, which is a limitation of our analysis.

## Discussion

Our results indicate that combined inhibition of AURKA and PAK1 is of potential value for the treatment of breast cancer, with greatest efficacy seen in luminal HR + and HER2 + subtypes in vitro. This could be explained by the interaction of AURKA and PAK1 with ERα (phosphorylation leading to ligand-independent activation), and with HER2 [4, 18]. AURKA promotes epithelial-mesenchymal transition and stem cell properties of ER + breast tumors in a mechanism involving overexpression of HER2 [39], while PAK1 is an essential mediator of HER2 signaling in mammary tumors dependent on this protein [13]. Correspondingly, our analysis of the METABRIC dataset showed significantly worse overall survival in patients with co-alterations of AURKA and PAK1, 2, or 3, with the greatest differences noted in patients with luminal A (HR +/HER2-) and B (HR +/HER2 +) tumors (Fig. S7).

The potency of the combination in luminal cell lines is likely due at least in part to the decreased phosphorylation of ERα at both the S305 and S118 residues, seen both in vitro and in xenograft experiments. Greater disruption of cell cycle control with the combination is also likely to contribute. In the BT474 xenograft model, the combination effectively inhibited signaling proteins linked to G1 and G2/M cell cycle control and ERα-activation, including cyclin B1, TFF1, C-MYC, and cyclin D1. This was consistent with the FACS analysis showing the combination arrested BT474 cells in both G1 and G2/M phases. One limitation of the present work is that we did not use cell sorting to separate mouse stromal cells from human breast cancer cells in these experiments; this may have led to somewhat diminished apparent effect of the drugs on phosphorylation of ERα.

We have expected a synergistic effect of alisertib and FRAX1036 on cell cycle and suppression of tumor growth because of more effective suppression of AURKA in the settings of PAK1 inhibition [15]. However, alisertib treatment also decreased phospho-PAK1/2/3, possibly via inhibition of phospho-AKT that can activate PAK1 [10, 38]. Notably, in silico analysis showed strong positive correlation of sensitivity to FRAX1036 with dependence on *AURKA* in ERα + tumors, providing a rationale to combine AURKA and PAK1-inhibitors.

The combination effectively inhibited expression of the transcription factor and proto-oncogene C-MYC, a protein frequently overexpressed in breast tumors, and implicated in poor clinical outcomes [36, 40]. Despite intense investigations, no effective strategies exist to target C-MYC. C-MYC upregulates the expression of AURKA [41], while AURKA activity protects C-MYC from degradation [42]. AURKA signals through C-MYC to induce telomerase, supporting tumor immortalization [43]. In kinase-independent functions, AURKA interacts with heterogeneous nuclear ribonucleoprotein K to activate *C*-*MYC* promoter, enhancing breast cancer stem cell phenotypes [44]. In parallel, C-MYC is as a downstream target of PAK1: PAK1 inhibition decreases C-MYC expression and signaling [12, 45]. Significant downregulation of C-MYC after combined treatment with AURKA and PAK1 inhibitors observed in our study is an exciting and clinically important finding. Our analysis of correlations with zGARP scores identified dependence on C-MYC as the strongest predictor of response to alisertib in ERα + lines. Luminal cell lines sensitive to *C*-*MYC* knock down required higher concentrations of alisertib for growth inhibition. While cell lines highly dependent on *C*-*MYC* have more compensatory mechanisms to escape alisertib-induced *C*-*MYC* downregulation, co-treatment with PAK1 inhibitors may abrogate these mechanisms, allowing response to lower doses of alisertib.

Together, our results provide evidence that dual inhibition of AURKA and PAK1 is of value in breast cancer. Enhanced anti-tumor activity of this combination is based on multiple mechanisms, including enhanced inhibition of phosphorylation of AURKA, PAK1, and ERα, as well as decreased expression of cell cycle proteins and C-MYC. Although resistance developed in vivo to single agent FRAX1036, addition of FRAX1036 to alisertib conferred significant advantages and lead to cases of complete or near complete tumor response, consistent with the concept that combination targeted therapy is beneficial because of synergistic anti-tumor effect and prevention of the selection of drug-resistant subclones during therapy [46].

One limitation of our study is that we examined the effects of the combination in a single in vivo model—further studies in PDXs and breast tumor cell organoids will be useful to confirm and extend our findings. In our study, as proof of concept, we used a prototype PAK1/2/3 inhibitor FRAX1036. Newer, more potent and selective PAK1 inhibitors now in development [47, 48] should be evaluated in combination with AURKA inhibitors in further studies. Alisertib was shown to be active in preclinical studies and early clinical trials in combination with microtubule inhibitors [7, 8, 49] or fulvestrant [50]. Given the findings of our study, evaluation of the combination of AURKA and PAK1 inhibitors together with other targeted or chemotherapeutic agents, such as tamoxifen, aromatase inhibitors, HER2-inhibitors, or taxanes, would be of interest. As genomic characterization of breast cancers becomes more advanced, understanding of the landscape of oncogenic drivers may help inform the optimal use of these valuable therapeutics.

## Electronic supplementary material

Below is the link to the electronic supplementary material.

Supplementary material 1 (DOCX 26 kb)

Supplementary material 2 (PPTX 8588 kb)

## Data Availability

The datasets analyzed during the study are publically available at [25–29].
