# Supplementary material for: Combined inhibition of Aurora A and p21-activated kinase 1 as a new treatment strategy in breast cancer
Source: Breast Cancer Res Treat. 2019 Jun 28;177(2):369–82. doi: 10.1007/s10549-019-05329-2 (PMC6661032; doi:10.1007/s10549-019-05329-2)
Supplement: Supplementary file 1 — Supplementary material 1 (DOCX 26 kb) [file 10549_2019_5329_MOESM1_ESM.docx]

**Combined inhibition of aurora A and p21-activated kinase 1 as a new treatment strategy in breast cancer**

Vlad A. Korben, Michelle Borakove, Yayi Feng, William M. Wuest, Alex B. Koval, Anna S. Nikonova, Ilya Serebriiskii, Jonathan Chernoff, Virginia F. Borges, Erica A. Golemis, and Elena Shagisultanova

**Supplementary Materials and Methods**

**Tumor cell lines, media and reagents.** Activity of alisertib and FRAX1036 as single agents and in combination was explored in 13 human breast tumor cell lines (5 luminal cell lines: MCF-7, T47D, ZR-75-1, BT474, MDA-MB-361; 4 HR-/HER2+ cell lines: SKBR3, HCC1954, HCC1419, HCC1569; and 4 TNBC cell lines: MDA-MB-231, MDA-MB-157, MDA-MB-468, HCC1806). MCF-7, HCC1806, MDA-MB-157 and MDA-MB-231 were cultured in DMEM; ZR-75-1, T47D, MDA-MB-361, HCC1954, HCC1419 and HCC1569 in RPMI; BT474, SKBR3 and MDA-MB-468 in DMEM/F12. Media was obtained from Corning Life Science (Corning*,* NY). Effects of Alisertib and FRAX1036 on cell signaling and cell cycle was studied in detail using the cell lines T47D (HR+/HER2- luminal A subtype) and BT474 (HR+/HER2+ luminal B subtype), because alisertib and FRAX1036 displayed the greatest synergy in the luminal and HER2+ tumor subtypes. BT474 was used for xenograft experiments to confirm *in vivo* effects of alisertib and FRAX1036 combination.

**Western Blotting.** We used primary antibodies for phospho-PAK1/2/3 (Thermo Fisher, Waltham, MA, #44-940G), phospho-ERα(S305) (EMD Millipore, Billerica, MA, #07-962), C-MYC (Abcam, Cambridge, MA, #32072), vinculin (Sigma-Aldrich, St. Louis, MO, #V9131), and GAPDH (Bio-Rad, Hercules, CA, #VMA00046); primary antibodies to PAK1 (#2602), ERα (#13258), phospho-ERα(S118) (#2511), AKT (#2920), phospho-AKT(T308) (#4056), PLK1 (#4513) and phospho-PLK1(Thr210) (#9062) were purchased from Cell Signaling (Danvers, MA).

**Xenograft studies.** Alisertib dissolved in 10% 2-hydroxypropyl-β-cyclodextrin (Sigma-Aldrich, St. Louis, MO) with 1% sodium bicarbonate was administered twice a day by oral gavage at 15 mg/kg as previously described [1-3]. FRAX1036 dissolved in 20% 2-hydroxypropyl-β-cyclodextrin in 50 mM citrate buffer (pH 3.0) was administered at 20mg/kg daily by oral gavage as previously described [4, 5]. Control group received vehicle solutions twice a day by oral gavage.

**Immunohistochemistry (IHC).** Ki-67 (#9027), cyclin D1 (#2978), TFF1 (#15571), and cleaved caspase-3 (#9664) antibodies were purchased from Cell Signaling (Danvers, MA). Cyclin B1 (#32053) and C-MYC (#32072) antibodies were purchased from Abcam (Cambridge, MA), phospho-AURKA T288 antibody (IHC-00067) was purchased from Bethyl Laboratories (Montgomery, TX).

**Statistical analysis.** Potential synergy of alisertib and FRAX1036 in tumor cell lines was eveluated by the Chou-Talalay Method [6]. Differences between treatment groups based on western blot and FACS analysis were analyzed using two-tailed t-test and one-way ANOVA with Dunnett’s multiple comparison test; we confirmed that all data were normally distributed. For *in vivo* experiments, differences in tumor growth curves were assessed by regression analysis (log relative ratio model); synergy was assessed by two-way and three-way interactions in a generalized linear model estimated by generalized estimating equations (GEE), assuming gaussian family, id link, and Markov working correlation matrix. Tumor volumes were compared using two-tailed t-test and one-way ANOVA with Dunnett’s multiple comparison test; we again confirmed data were normally distributed. Differences between treatment groups observed from quantitative immunohistochemistry were evaluated by Mann-Whitney test and Kruskal Wallis analysis with Dunn’s multiple comparison test (non-parametric data). Pearson correlation analysis was used to evaluate correlation between expression data or z-GARP scores and activity of alisertib and FRAX1036 in tumor cell lines. Results of one-way ANOVA / Dunnett’s test and Kruskal-Wallis analysis / Dunn’s test are summarized in Table S1 (supplemental data).

**References**

1. Manfredi MG, Ecsedy JA, Chakravarty A, Silverman L, Zhang M, Hoar KM, Stroud SG, Chen W, Shinde V, Huck JJ *et al*: **Characterization of Alisertib (MLN8237), an investigational small-molecule inhibitor of aurora A kinase using novel in vivo pharmacodynamic assays**. *Clinical cancer research : an official journal of the American Association for Cancer Research* 2011, **17**(24):7614-7624.

2. Manfredi MG, Ecsedy JA, Meetze KA, Balani SK, Burenkova O, Chen W, Galvin KM, Hoar KM, Huck JJ, LeRoy PJ *et al*: **Antitumor activity of MLN8054, an orally active small-molecule inhibitor of Aurora A kinase**. *Proceedings of the National Academy of Sciences of the United States of America* 2007, **104**(10):4106-4111.

3. Yang JJ, Li Y, Chakravarty A, Lu C, Xia CQ, Chen S, Pusalkar S, Zhang M, Ecsedy J, Manfredi MG *et al*: **Preclinical drug metabolism and pharmacokinetics, and prediction of human pharmacokinetics and efficacious dose of the investigational Aurora A kinase inhibitor alisertib (MLN8237)**. *Drug Metab Lett* 2014, **7**(2):96-104.

4. Prudnikova TY, Villamar-Cruz O, Rawat SJ, Cai KQ, Chernoff J: **Effects of p21-activated kinase 1 inhibition on 11q13-amplified ovarian cancer cells**. *Oncogene* 2016, **35**(17):2178-2185.

5. Prudnikova TY, Chernoff J: **The Group I Pak inhibitor Frax-1036 sensitizes 11q13-amplified ovarian cancer cells to the cytotoxic effects of Rottlerin**. *Small GTPases* 2016:1-6.

6. Chou TC, Talalay P: **Quantitative analysis of dose-effect relationships: the combined effects of multiple drugs or enzyme inhibitors**. *Advances in enzyme regulation* 1984, **22**:27-55.
