# Supplementary material for: Combined inhibition of Aurora A and p21-activated kinase 1 as a new treatment strategy in breast cancer
Source: Breast Cancer Res Treat. 2019 Jun 28;177(2):369–82. doi: 10.1007/s10549-019-05329-2 (PMC6661032; doi:10.1007/s10549-019-05329-2)
Supplement: Supplementary file 2 — Supplementary material 2 (PPTX 8588 kb) [file 10549_2019_5329_MOESM2_ESM.pptx]

## Slide 1
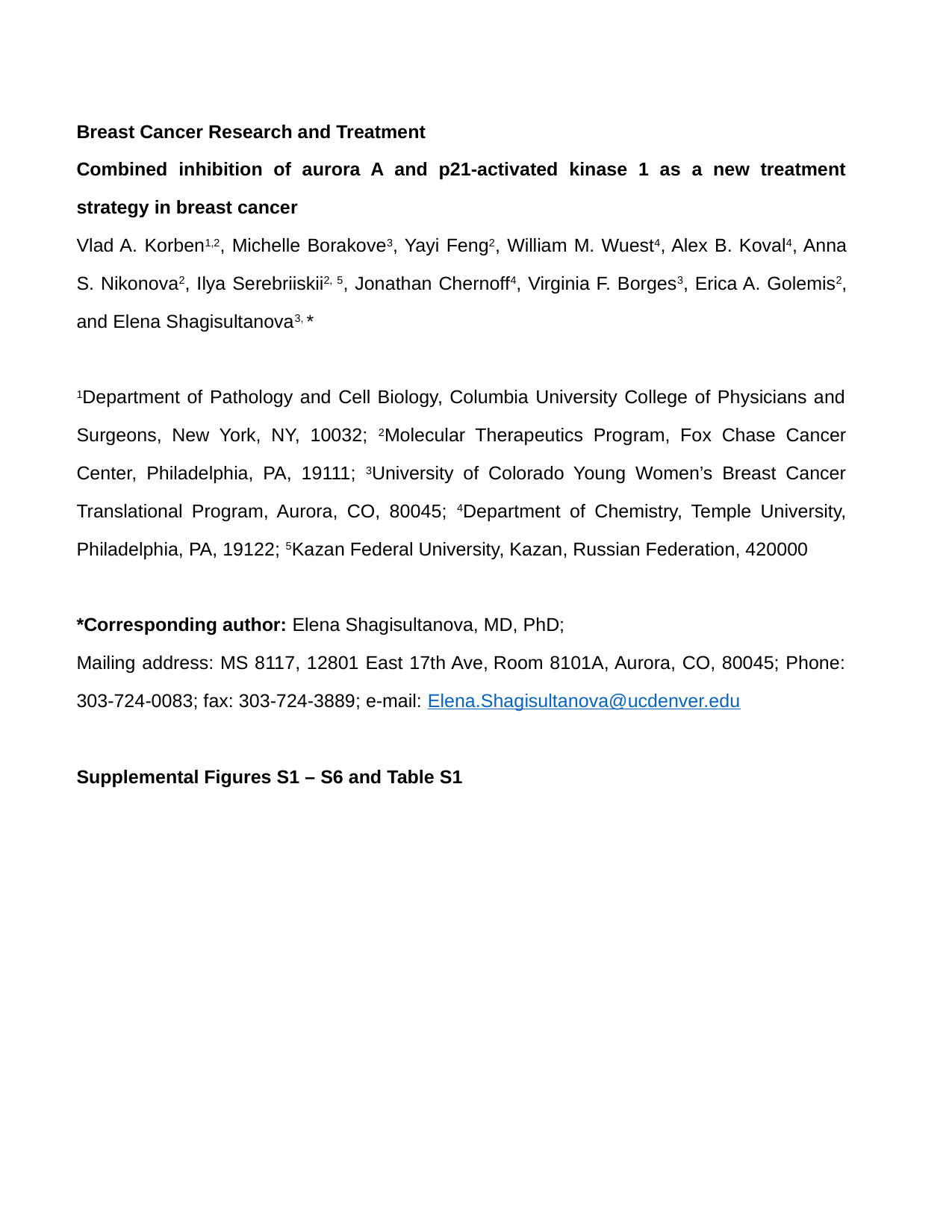

Breast Cancer Research and Treatment
Combined inhibition of aurora A and p21-activated kinase 1 as a new treatment strategy in breast cancer
Vlad A. Korben1,2, Michelle Borakove3, Yayi Feng2, William M. Wuest4, Alex B. Koval4, Anna S. Nikonova2, Ilya Serebriiskii2, 5, Jonathan Chernoff4, Virginia F. Borges3, Erica A. Golemis2, and Elena Shagisultanova3, *
1Department of Pathology and Cell Biology, Columbia University College of Physicians and Surgeons, New York, NY, 10032; 2Molecular Therapeutics Program, Fox Chase Cancer Center, Philadelphia, PA, 19111; 3University of Colorado Young Women’s Breast Cancer Translational Program, Aurora, CO, 80045; 4Department of Chemistry, Temple University, Philadelphia, PA, 19122; 5Kazan Federal University, Kazan, Russian Federation, 420000
*Corresponding author: Elena Shagisultanova, MD, PhD;
Mailing address: MS 8117, 12801 East 17th Ave, Room 8101A, Aurora, CO, 80045; Phone: 303-724-0083; fax: 303-724-3889; e-mail: Elena.Shagisultanova@ucdenver.edu
Supplemental Figures S1 – S6 and Table S1

## Slide 2
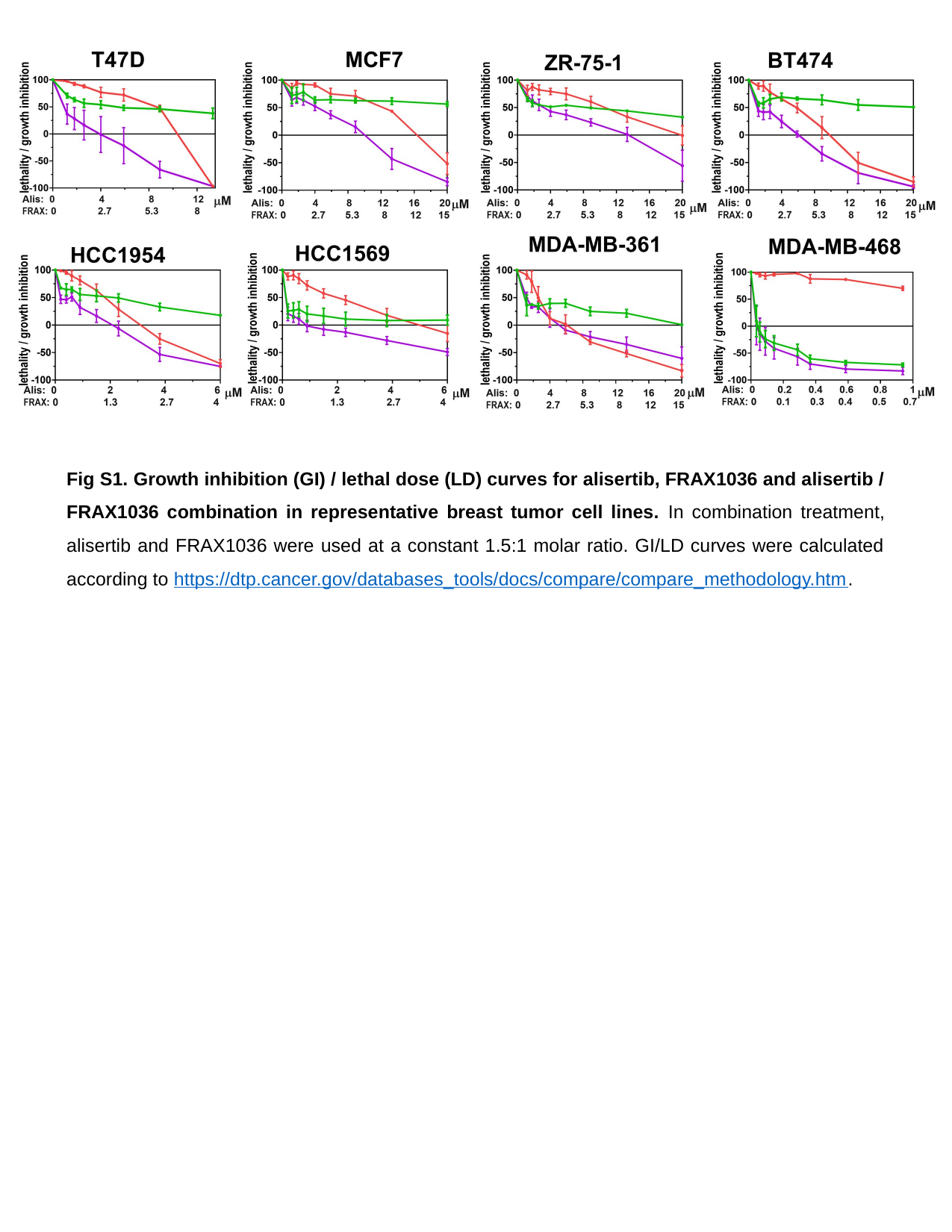

Fig S1. Growth inhibition (GI) / lethal dose (LD) curves for alisertib, FRAX1036 and alisertib / FRAX1036 combination in representative breast tumor cell lines. In combination treatment, alisertib and FRAX1036 were used at a constant 1.5:1 molar ratio. GI/LD curves were calculated according to https://dtp.cancer.gov/databases_tools/docs/compare/compare_methodology.htm.

## Slide 3
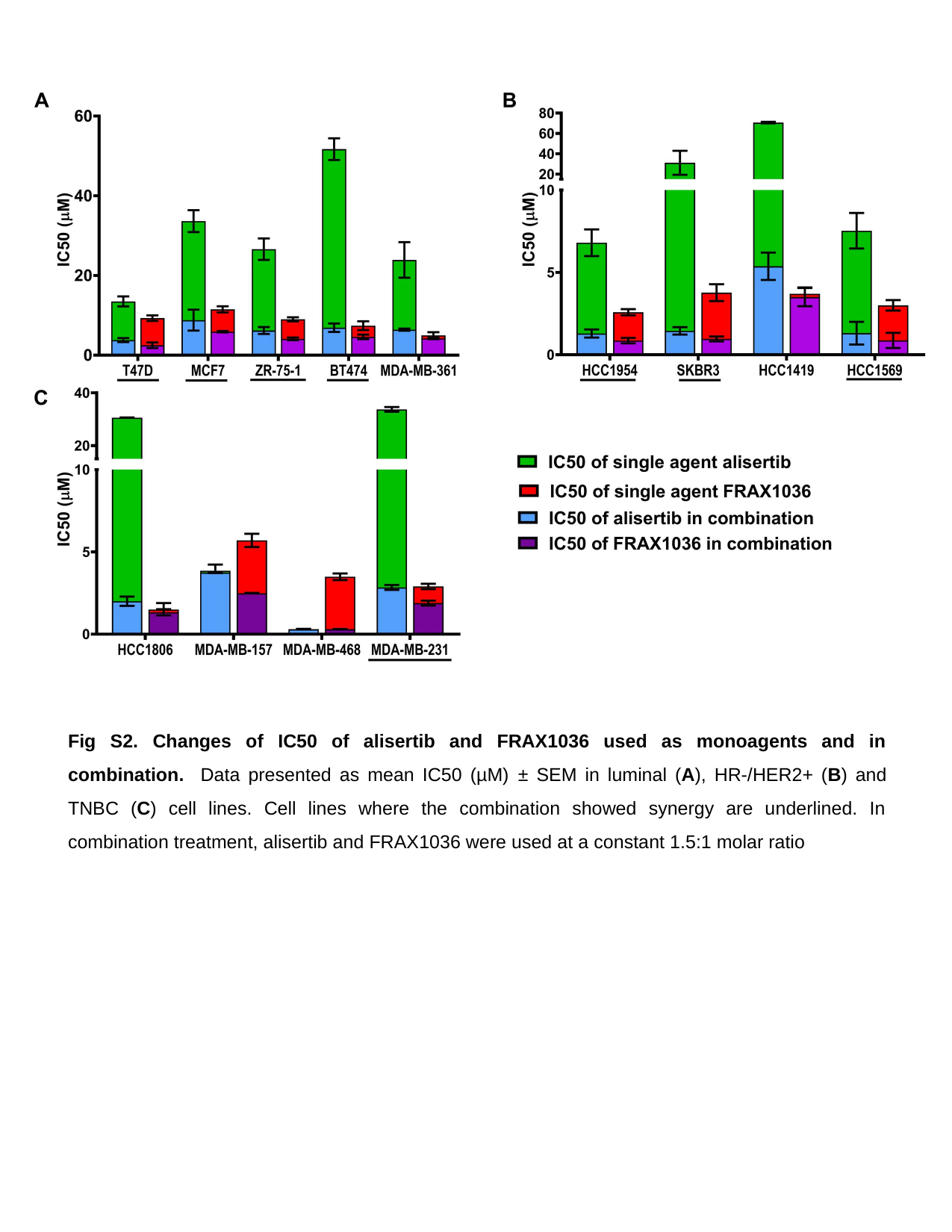

Fig S2. Changes of IC50 of alisertib and FRAX1036 used as monoagents and in combination. Data presented as mean IC50 (µM) ± SEM in luminal (A), HR-/HER2+ (B) and TNBC (C) cell lines. Cell lines where the combination showed synergy are underlined. In combination treatment, alisertib and FRAX1036 were used at a constant 1.5:1 molar ratio

## Slide 4
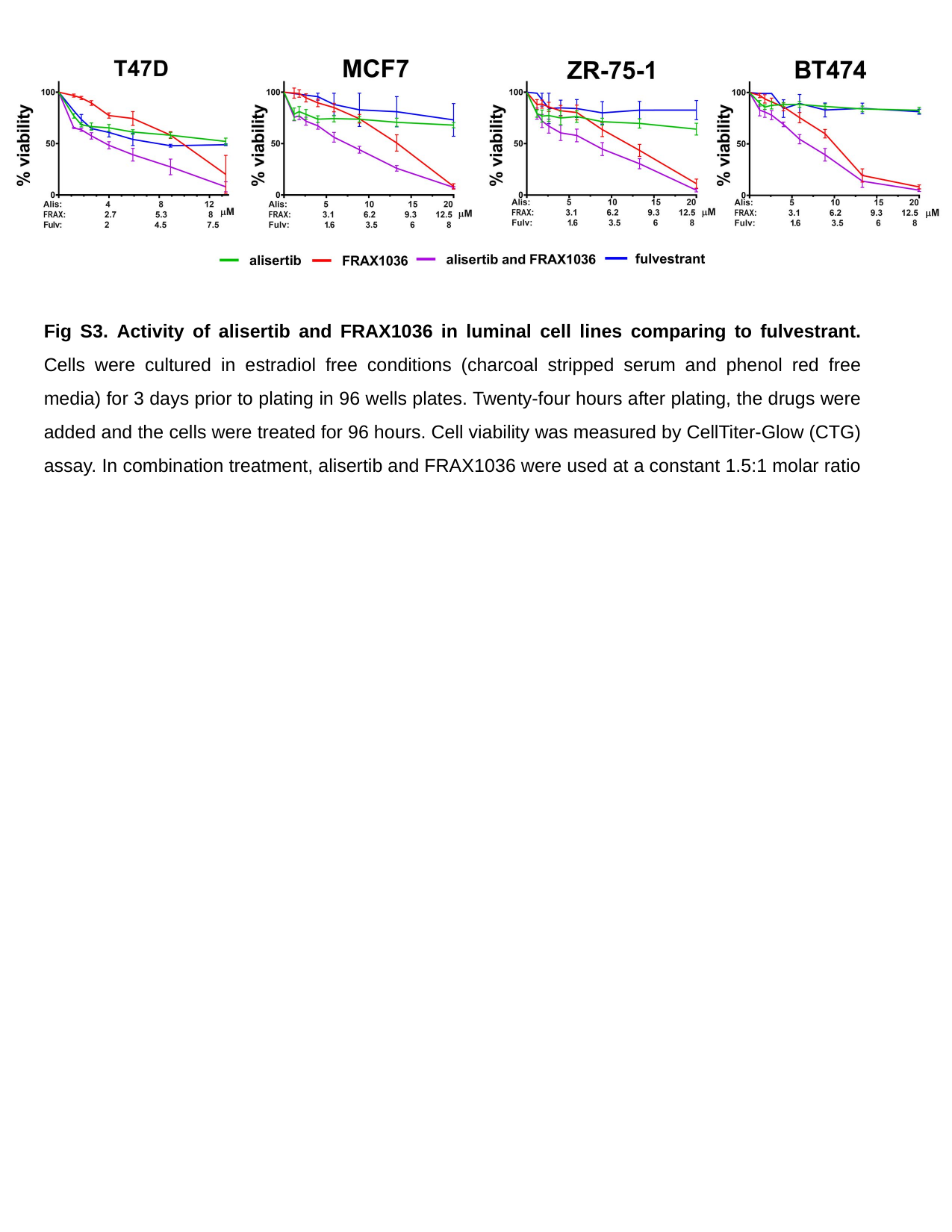

Fig S3. Activity of alisertib and FRAX1036 in luminal cell lines comparing to fulvestrant. Cells were cultured in estradiol free conditions (charcoal stripped serum and phenol red free media) for 3 days prior to plating in 96 wells plates. Twenty-four hours after plating, the drugs were added and the cells were treated for 96 hours. Cell viability was measured by CellTiter-Glow (CTG) assay. In combination treatment, alisertib and FRAX1036 were used at a constant 1.5:1 molar ratio

## Slide 5
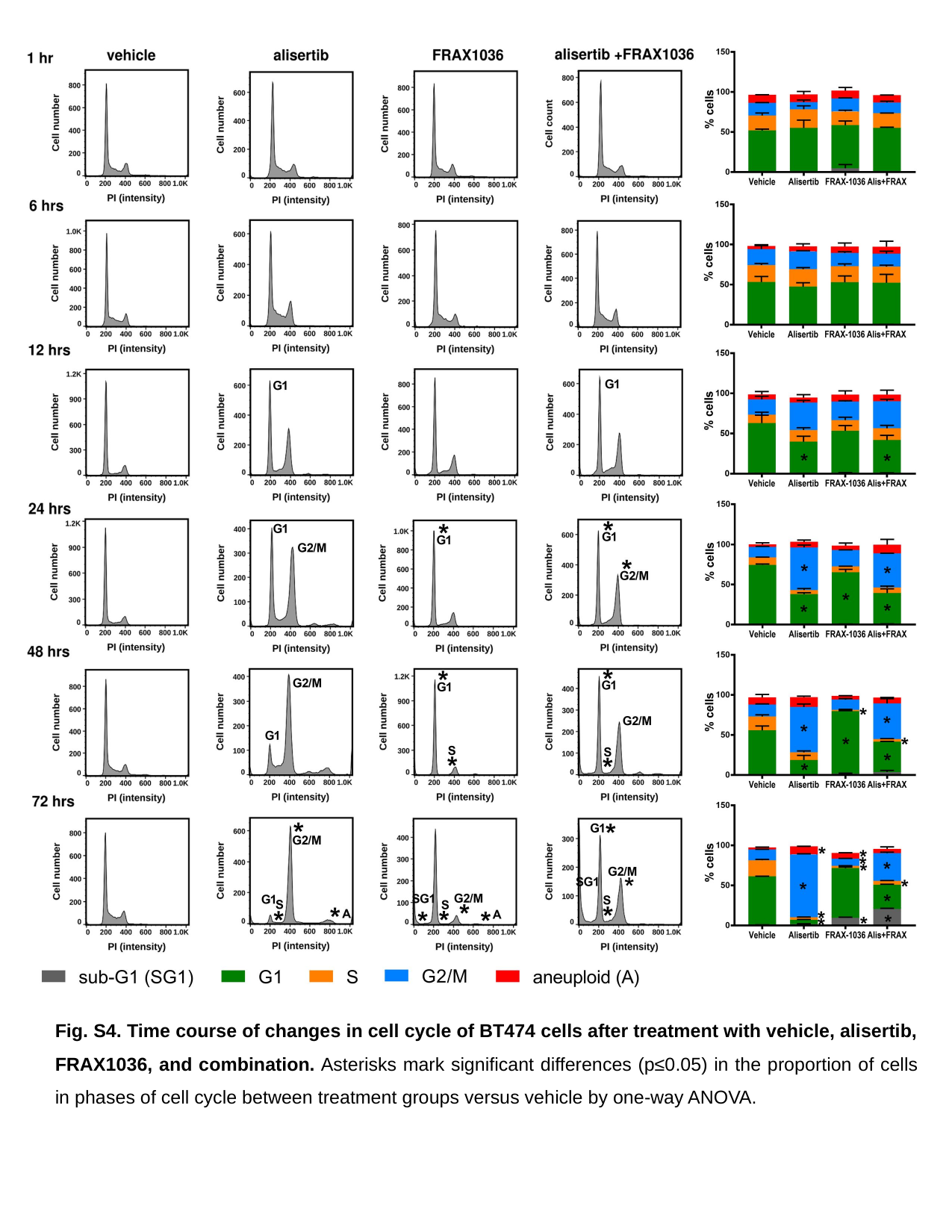

Fig. S4. Time course of changes in cell cycle of BT474 cells after treatment with vehicle, alisertib, FRAX1036, and combination. Asterisks mark significant differences (p≤0.05) in the proportion of cells in phases of cell cycle between treatment groups versus vehicle by one-way ANOVA.

## Slide 6
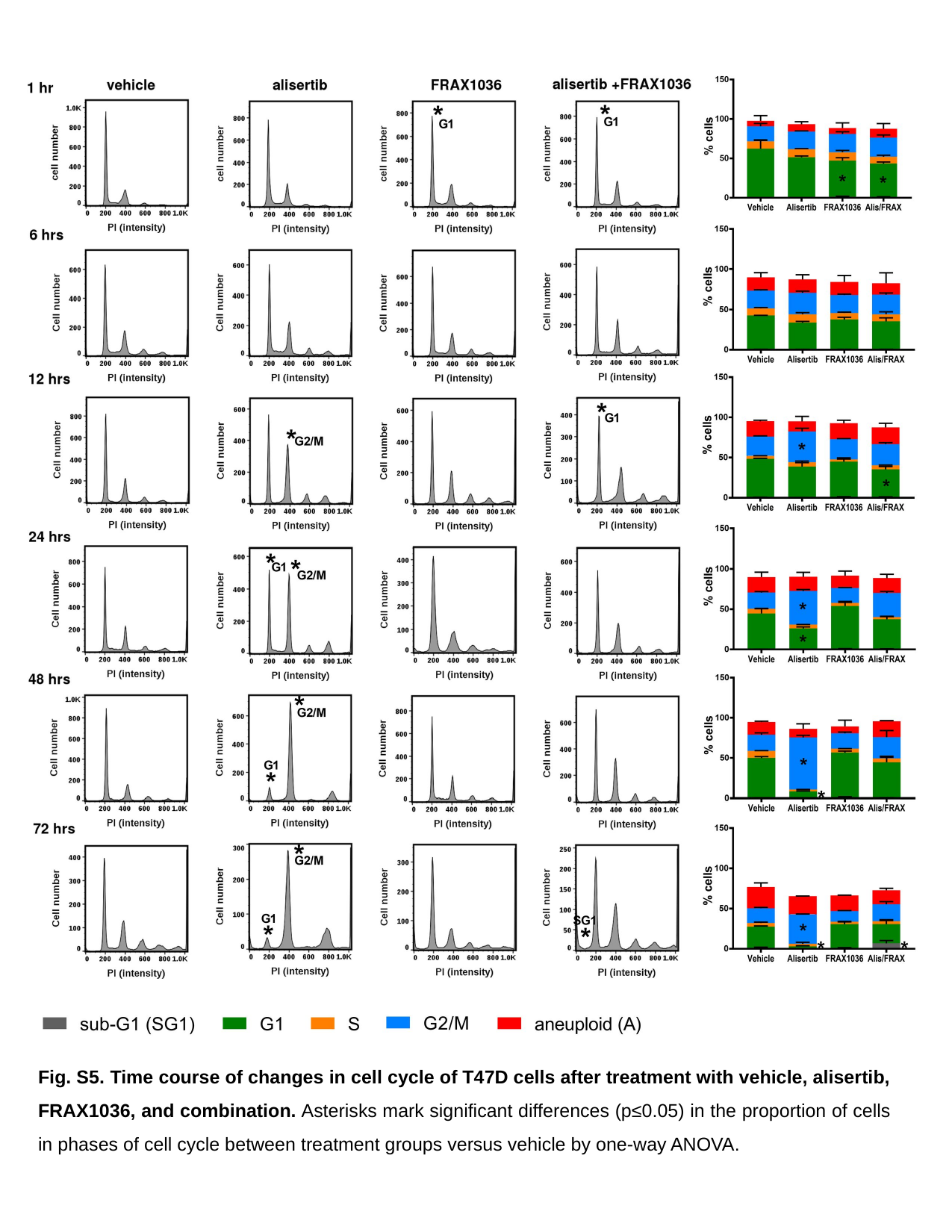

Fig. S5. Time course of changes in cell cycle of T47D cells after treatment with vehicle, alisertib, FRAX1036, and combination. Asterisks mark significant differences (p≤0.05) in the proportion of cells in phases of cell cycle between treatment groups versus vehicle by one-way ANOVA.

## Slide 7
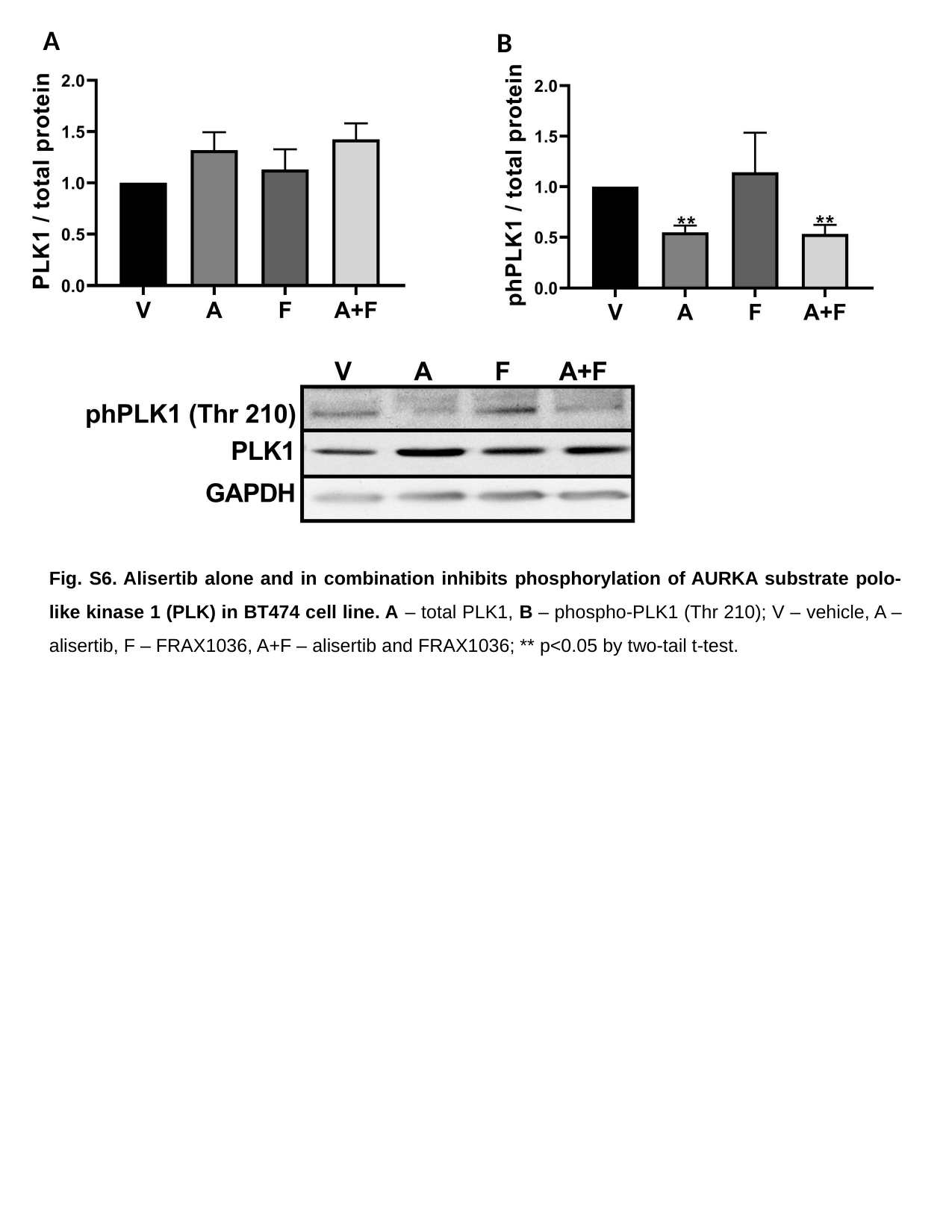

A
B
Fig. S6. Alisertib alone and in combination inhibits phosphorylation of AURKA substrate polo-like kinase 1 (PLK) in BT474 cell line. A – total PLK1, B – phospho-PLK1 (Thr 210); V – vehicle, A – alisertib, F – FRAX1036, A+F – alisertib and FRAX1036; ** p<0.05 by two-tail t-test.

## Slide 8
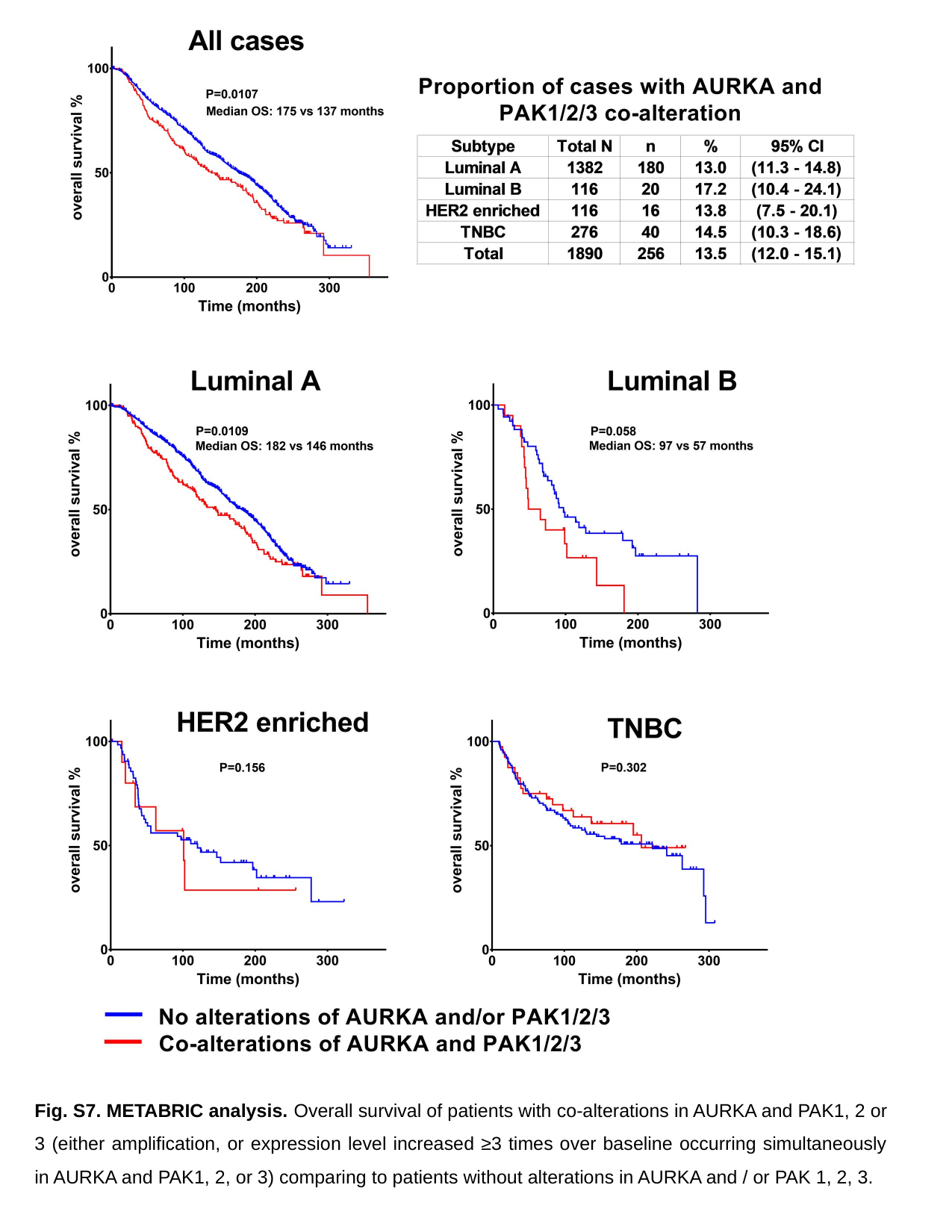

Fig. S7. METABRIC analysis. Overall survival of patients with co-alterations in AURKA and PAK1, 2 or 3 (either amplification, or expression level increased ≥3 times over baseline occurring simultaneously in AURKA and PAK1, 2, or 3) comparing to patients without alterations in AURKA and / or PAK 1, 2, 3.

## Slide 9
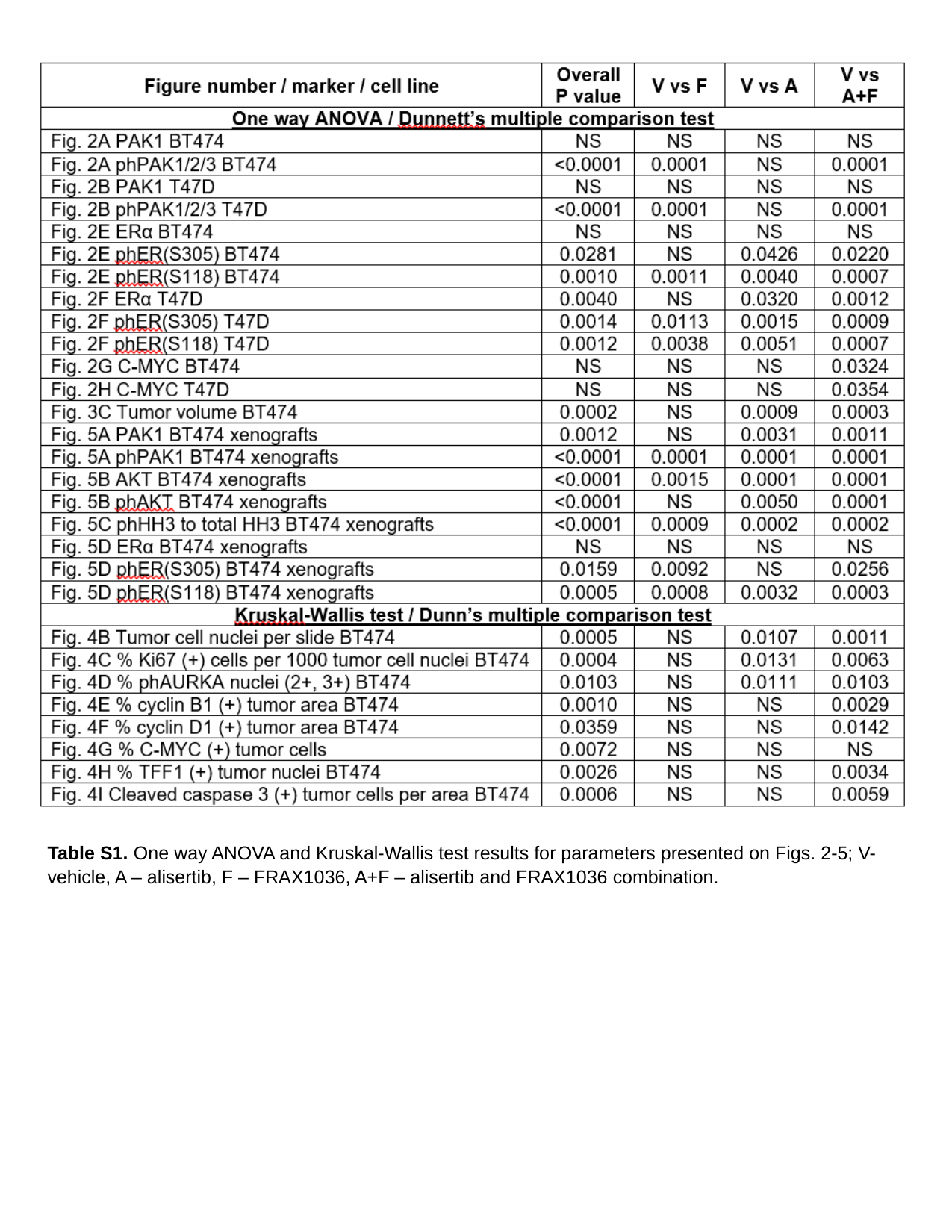

Table S1. One way ANOVA and Kruskal-Wallis test results for parameters presented on Figs. 2-5; V- vehicle, A – alisertib, F – FRAX1036, A+F – alisertib and FRAX1036 combination.
